# Gait Analysis, Metabolic Parameters and Adherence to the Mediterranean Diet in Patients with Type 2 Diabetes Mellitus Compared with Healthy Controls: A Pilot Study

**DOI:** 10.3390/nu15153421

**Published:** 2023-08-02

**Authors:** Dimitris Efthymiou, Niki Katsiki, Dimitrios Xipolias Zekakos, Panagiotis Vassiliadis, Alexandros Petrelis, Emilia Vassilopoulou

**Affiliations:** 1 Nous Therapy Center, 1 Aggelaki Street, 54621 Thessaloniki, Greece; dimitrisefthy@gmail.com; 2 Department of Nutritional Sciences and Dietetics, International Hellenic University, 57400 Thessaloniki, Greece; nikikatsiki@hotmail.com; 3 School of Medicine, European University Cyprus, 2404 Nicosia, Cyprus; 4 Digitsole SAS, 54000 Nancy, France; dimxyp@gmail.com (D.X.Z.); apetrelis@live.com (A.P.); 5 Papanikolaou General Hospital-Psychiatric Hospital, 56430 Thessaloniki, Greece; vaslop@otenet.gr; 6 Pediatric Unit, Fondazione IRCCS Ca’ Granda Ospedale Maggiore Policlinico, 20122 Milan, Italy

**Keywords:** type 2 diabetes mellitus, peripheral neuropathy, gait analysis, diabetes distress, MedDiet score

## Abstract

Background and purpose: Patients with type 2 diabetes mellitus (T2DM) are prone to developing diabetic peripheral neuropathy (DPN) with an increased risk of injuries while walking, potentially leading to plantar ulcers. We aimed to assess the early gait changes in T2DM patients without clinical signs of DPN in comparison to age-matched healthy controls (HC). Subjects and Methods: One hundred T2DM patients (78 women, mean age: 66.4 ± 11.5 years) and 50 age-matched HC (34 women, mean age 62.1 ± 7.9 years) were evaluated with the PODOSmart^®^ gait analysis device. Anthropometric and biochemical data, as well as dietary habits were collected for all participants. T2DM patients also completed the Diabetes Distress (DS) self-report validated questionnaire. Results: One patient was excluded from the study due to lack of recent biochemical data. Among the T2DM patients, 88.9% reported little or no DS and 11.1% moderate DS. The T2DM group had higher body mass index, waist circumference, systolic blood pressure, glycated hemoglobin A1c, sodium, white blood cell count, triglycerides and low-density lipoprotein cholesterol, but lower high-density lipoprotein cholesterol than HC (*p* < 0.05 for all comparisons). The MedDiet score was satisfactory in both groups (*p* > 0.05). Significant differences were found between the two study groups in gaitline heel off, propulsion speed, foot progression angle, time taligrade phase, stride length, walking speed, angle attack, oscillation speed, pronation-supination toe off and clearance. Conclusions: The T2DM patients without self-reported DS or clinical signs of DPN may exhibit significant differences in several gait parameters analyzed with PODOSmart^®^. Whether gait analysis can be used as an early diagnostic tool of T2DM complications should be further explored.

## 1. Introduction

Type 2 diabetes mellitus (T2DM) is characterized by chronic hyperglycemia, resulting in significant long-term complications for various tissues and organs, such as eyes, kidneys, nerves and blood vessels [1]. Among diabetic microvascular complications, diabetic peripheral neuropathy (DPN) usually initiates as a sensor type disorder and progresses into a kinetic type of neuropathy that results in muscle atrophies and reduced joint mobility, thus affecting gait [2]. Almost half of people with type 1 DM and T2DM have either neuropathic symptoms or conduction disorders, whereas the reported incidence of DPN ranges between 6 and 51% depending on age, glucose control, duration and type of diabetes [3]. Other risk factors for DPN include obesity, metabolic disorders and micro RNAs [4]. Of note, DPN can predispose to the incidence of several complications of the lower extremities, including foot ulcers and infections, chronic pain and amputations [3]. With regard to nerve damage, larger diameter nerve fibers are more vulnerable than smaller ones; thus, the fine touch sensation is the first to be disturbed, followed by pain and increased local temperature [5]. There is a hypothesis that the abnormal load in foot structures that are normally not charged can lead to foot ulcers and an increased risk of amputations [5].

Human gait involves a complex interaction of the musculoskeletal, nervous and cardiorespiratory systems and is influenced by age, mood, personality and sociocultural status [6]. Gait impairment can lead to falls, injuries and loss of personal freedom, thus significantly reducing an individual’s quality of life [6]. Clinical gait analysis is subjective, being influenced by the observer’s expertise, and thus, quantitative instrumented gait analysis was developed to provide accurate and reliable gait data to clinicians [7].

In DM patients, sensory impairment (presence of impaired vibration and protective sensation and/or neuropathic ulcer), reduced lower-extremity strength and central nervous system dysfunction may contribute to the development of gait disorders [8]. It has been suggested that impaired gait performance may be linked to the early stages of DPN in T2DM patients without clinical symptoms and signs of DPN [9]. Although gait abnormalities occur across the diabetes spectrum and worsen with disease severity, large fiber neuropathy occurs as an early silent symptom [10], and gait abnormalities rise with diabetes severity [11].

In general, an older person’s preferred walking speed is a sensitive indicator of their general health and likelihood of survival. Executive control and sound knowledge are necessary for safe walking. Gait abnormalities significantly lower quality of life and cause loss of personal freedom, falls and injuries [6]. The inclusion criteria, neuropathy definition, gait analysis surface, data collection techniques and sensor technologies employed in gait observational and gait analysis studies of DM patients show substantial variability. As a result, the findings are not always definitive. 

However, by paying attention to how people with diabetes walk, useful information might be gleaned in order to prevent complications [12]. Patients with DM have slower walking speeds and walking paces (measured as the distance between the heels, perpendicular to the progress bar). The higher incidence of foot ulcers and these two early symptoms of DPN can also be used as warning indications [13]. Additionally, they demonstrate a decrease in knee mobility, pitch variability, maximal vertical reactive ground force component, plantar flexion moment and stride length [14]. There are also longer support times for the double limbs and longer gait cycle completion times [14]. 

At slower walking speeds, the majority of these additional characteristics might be irrelevant. Reduced ground reaction forces, decreased joint angles, decreased one-limb support and increased double-limb support time are all related to slower walking speeds. According to Raspovic’s (2013) study [15], patients with a history of ulcers had significantly less ankle and early metatarsophalangeal joint range of motion. Patients with diabetes frequently experience a functional or structural hallux limit along with a reversal of the sagittal joint’s movement. According to Dananberg, this pattern of gait modifications comprises reduced big toe dorsiflexion during propulsion, delayed plantar flexion of the ankle (heel lift), delayed or failed extension of the knee, extension of the knee and reduction of big toe dorsiflexion [16]. 

Additionally, older T2DM patients who follow the Mediterranean diet (MedDiet) had better gait characteristics [17]. In particular, McClure et al. found that older persons with T2DM who consumed more fish and shellfish had improved gait speed [17]. 

In the current study, we aimed to investigate early gait alterations in T2DM patients without clinical signs and symptoms of DPN in comparison to healthy individuals, taking also into consideration adherence to MedDiet and diabetes distress (DS).

## 2. Materials and Methods

Patients diagnosed with T2DM based on the World Health Organization (WHO) diagnostic criteria (Classification of diabetes mellitus. Geneva: World Health Organization; 2019. Licence: CC BY-NC-SA 3.0 IGO) and regularly monitored (on a 3–6 months basis) at the Pathological Clinic of the George Papanikolaou Hospital were recruited from March to December 2021. DPN was excluded during clinical assessment with the Michigan Neuropathy Screening Instrument (MNSI) scale, which includes a questionnaire and bipedal physical examination [18]. The inclusion criteria were: (1) age >18 years, (2) diagnosis of T2DM and (3) no signs/symptoms of DPN. For the control group, age-matched healthy individuals that visited the clinic for their regular health monitoring during the study period but were not diagnosed with T2DM (representing the healthy controls, HC) were recruited.

The presence of one or more of the following criteria for both T2DM patients and HC were a reason for exclusion: (1) diagnosis, symptoms or signs of DPN, (2) inability to walk independently, (3) history of lower-extremity amputation (toe), (4) obvious foot deformity, (5) lower limb ulcer, (6) brain injury, (7) poor vision affecting walking, (8) various spinal diseases, (9) severe liver and kidney impairment, (10) pregnancy or (11) diabetes acute complications.

Before inclusion in the study, all participants were informed in detail about the study’s aim and protocol, and they provided their written informed consent. The study was approved by the Research Ethics Committee of the Aristotle University of Thessaloniki (code number 4.307/26 January 2021) and complied with the International Code of Medical Ethics of the World Medical Association and the Helsinki Declaration.

### 2.1. Adherence to MedDiet

Adherence to the MedDiet was assessed with the MedDiet Score questionnaire, which recorded consumption of the 11 major components (unrefined grains, fruits, vegetables, potatoes, legumes, olive oil, fish, red meat, poultry, full-fat dairy products and alcohol) in a face-to-face interview with a qualified dietitian. The MedDiet score is categorized as no adherence (0–13), inadequate adherence (14–27), satisfactory adherence (28–41), and very good adherence (42–55) [19].

### 2.2. Anthropometric Data

Anthropometric measurements were taken by a trained investigator in the morning, after at least 8 h of fasting, immediately before gait analysis. Body height was measured to the nearest 0.1 cm using a commercially available stadiometer (Leicester Height Measure, Invicta Plastics Ltd., Oadby, UK) with participants walking barefoot, shoulders relaxed, arms hanging freely and head in the horizontal Frankfort plane. Participants were weighed barefoot and in light clothing to the nearest 0.1 kg using a TANITA RD-545 (“RD-545-Connected smart scale | Tanita Official Store,” n.d.). Body mass index (BMI) was calculated as follows: Weight (kg) by height squared (m^2^). Waist circumference (WC) was measured with a flexible, nonstretch SECA measuring tape to an accuracy of 1 mm on a horizontal plane after exhalation at a point equidistant from the lowest mobile rib and the upper edge of the iliac crest.

### 2.3. Diabetes Distress Scale

The Diabetes Distress Scale (DDS) has been used to assess disease burden in T2DM patients [20]. The DDS consists of 17 items with four subscales related to the four major domains that define diabetes-related distress (DRD): emotional distress (EB) (5 items), physician-related distress (PD) (4 items), regime-related distress (RD) (5 items) and interpersonal distress (ID) (3 items). Responses to each item are based on a 6-point Likert scale and rated from 1 (“no problem”) to 6 (“a very serious problem”) during the past month. The overall item mean is calculated by summing the responses to all items and dividing by 17. The mean of each subscale is calculated by summing the responses to all items in that subscale and dividing by the respective number of items. A score of <2.0 is considered “little or no distress”, 2.0–2.9 is considered “moderate distress” and ≥3.0 is considered “severe distress”. The “clinically significant” value for DRD is set at ≥2.0.

### 2.4. Biochemical Data

Venous blood samples were collected from all participants as part of routine monitoring on a day independent of gait analysis after they had fasted overnight and analyzed on hospital premises using automated biochemical analyzers under standard conditions. Specifically, white blood cell count (WBC), blood glucose (GL), serum total cholesterol, triglycerides (TG), high-density lipoprotein cholesterol (HDL-C), high-sensitivity C-reactive protein (hsCRP) and B12 were measured with an automatic analyzer (Toshiba TBA 120FR; Toshiba Medical Systems Co., Ltd., Tokyo, Japan). Low-density lipoprotein cholesterol (LDL-C) was calculated according to the Friedewald equation [21].

### 2.5. Gait Analysis

The PODOSmart^®^ gait analyzer was used to measure walking and running parameters in all participants. The PODOSmart^®^ insoles (Digitsole SAS, Nancy, France) consist of a pair of insoles (available in most shoe sizes) connected to a mobile application via a Bluetooth connection box. Rechargeable via USB, PODOSmart^®^ insoles can be used for more than 33 h. Each PODOSmart^®^ insole contains an inertial platform that records the position of each foot and walking or running steps in 3D space. The Bluetooth connection box collects all the data collected by the smart insoles. Then, all data are processed by artificial intelligence algorithms into a clinically useful dataset to extract spatiotemporal, kinematic and biomarker parameters, which are displayed on the user interface [22]. 

Both monopedal and bipedal gait data were recorded. Monopedal data included the angles of the foot during heel off (HO), heel strike (HS), toe off (TO) and flat foot in (FFI), the stride length in meters, stride duration in milliseconds, stance time, swing time and foot progression angle in degrees. Bipedal data included 3 gait parameters, i.e., cadence, gait speed (km/h) and double contact duration (%). More details on the gait analysis can be found in a previous study [23].

Participants removed their shoes and wiped their feet with alcohol. A member of the study team placed a PODOSmart^®^ insole in each shoe. Each participant walked comfortably straight ahead on level ground for 60 s and was then asked to turn around halfway and return to the starting line.

### 2.6. Statistical Analysis

Statistical analysis was performed with the R software (version 4.04) and R studio (version 1.4.1106, Boston, MA, USA). Data were reported as mean (±standard deviation, SD) or median (median absolute deviance, MAD [24]. Due to the squaring of deviations from the mean in its calculation, the SD is a measure that is greatly influenced by outliers and extreme values. Therefore, for normally distributed data, we utilized SD. We employed MAD as a measure of dispersion because SD is less trustworthy for skewed or strongly tailed distributions, where such outliers or extreme values are more prevalent. Because MAD determines the average absolute deviation from the mean, it is less susceptible to outliers. Thus, in order to analyze this dataset and provide unbiased insight into the nature of our data, the MAD was used; it provides a more “robust” measure of dispersion for data that does not follow a normal distribution. Normality was checked using the Shapiro–Wilk test. Normally distributed numerical data were compared by Student’s *t*-test and parametrical data by the Wilcoxon test. 

To further investigate the associations between gait analysis parameters and anthropometric and biochemical data, the Random Forest machine learning model was performed. It is a powerful machine learning algorithm that combines predictions from multiple machine learning algorithms (decision trees) and is widely used for both regression and classification tasks due to its robustness, simplicity, versatility and accuracy. As an ensemble learning method, it combines predictions from multiple machine learning algorithms to make a more accurate prediction than any single model. In the case of Random Forest, the base learners are decision trees.

A decision tree is a simple but effective machine learning model that follows a tree structure. Each internal node of the tree corresponds to a feature in the data, each branch represents a rule or decision and each leaf node represents an outcome. However, the main problem with decision trees is that they tend to overfit, i.e., they fit the training data too well and perform poorly on unseen data.

Each decision tree in the forest is trained on a different subset of the training data. This subset is obtained by sampling with replacement from the original data, a process also known as bootstrapping. Mathematically, if we have N instances in our original dataset, we still have N instances in our bootstrap sample, but because of replacement, some instances may appear more than once and others may not appear at all. 

The learning process of a decision tree is as follows: in a standard decision tree, the best feature for partitioning is selected by reducing a given criterion. In a classification problem, this criterion is often the Gini impurity or entropy, whereas in a regression problem, it is typically the variance. For example, the Gini impurity for a group of elements with J classes is calculated as follows:

Gini Impurity = 1 − ∑ (p i)^2^ for i = 1 to J,

where p i is the proportion of items labeled with class I in the set.

However, in a Random Forest, only a random subset of the features at each node is used for partitioning, instead of using all features. This subset contains m features, where m is typically sqrt(p) for classification and p/3 for regression, where p is the total number of features. This introduces further randomness into the model, making the individual trees less correlated and thus, increasing the robustness of the model. After all trees have been trained, the predictions of all trees are added together when making predictions with the Random Forest. In a classification problem, the final prediction is the most frequent prediction (mode) among all trees. In a regression problem, it is the average prediction (mean).

Finally, Random Forest provides a built-in measure of feature importance. This is done by calculating the average reduction in the criterion (Gini pollution, entropy or variance) that results from the split over a given feature, averaged over all trees in the forest. The larger the reduction, the more important the feature is ranked. RF addresses the problem of overfitting by creating a large number of decision trees at training time and outputting the class representing the mode of the classes in classification, or the mean prediction of the individual trees in regression. The key idea behind Random Forest is the law of large numbers—by averaging or combining many imperfect trees, we can create a model that is much more accurate and robust than any of its individual components.

RF introduces randomness into the model building process, further increasing the diversity and robustness of the model. This randomness comes into play in two ways. First, each tree of the forest is trained on a different subset of the training data, which is replaced by a random sample (also known as bootstrapping). Second, rather than finding the best feature for the split as in a normal decision tree, the random forest considers only a random subset of features for each split. This random subset of features helps to make the decision trees less correlated, making the ensemble model more robust.

In addition, random forest models are relatively easy to use because they require very few hyperparameters and are relatively insensitive to the values to which they are set. They also have built-in feature importance measures that make them very useful for interpretability.

In summary, Random Forest is a powerful and versatile machine learning method that combines multiple decision trees to create a model that is often superior in terms of predictive accuracy and robustness [25].

A 2-sided *p*-value of <0.05 was considered statistically significant. 

## 3. Results

From the 100 T2DM patients initially recruited in the study, one was excluded due to unresponsiveness to performing the appropriate biochemical tests. Table 1 summarizes the demographic, clinical and biochemical characteristics of the study groups. The mean age of the T2DM and HC groups were 66.4 (±11.5) and 62.1 (±7.9) years (*p* = 0.657). The T2DM patients had significantly higher weight (*p* < 0.001), BMI (*p* < 0.001), WC (*p* < 0.001), SBP (*p* < 0.001), glucose (*p* < 0.001), HbA1c (*p* < 0.001), triglycerides (*p* = 0.005), WBC (*p* < 0.001), uric acid (*p* < 0.001), urea (<0.001) and creatinine (*p* = 0.002), as well as lower HDL-C (*p* < 0.001). On the other hand, they presented significantly lower total cholesterol (*p* = 0.002) and LDL-C (*p* = 0.007) than the HC.

Furthermore, T2DM patients presented a higher percentage of comorbidities compared with HC: 8 vs 2.5% for hypothyroidism, 40 vs 5% for hypertension/ arrhythmia, 35 vs. 2% for hypolipidemic drugs and 3 vs 0% for anti-acid therapy, respectively.

Individual dietary habits as well as the MedDiet Score did not differ among the two study groups (Table 2). Both T2DM and HC participants had a satisfactory adherence to the MedDiet (mean MedDiet Score 34 for both groups). We could not identify significant differences in any of the Mediterranean Diet Score items, revealing the similar dietary profile of the two groups.

With regard to DDS, 88% of the T2DM patients had mean scores lower than 2 in all DDS subscales (mean total score 1.1), whereas 11% had a moderate distress (total score 2.2). 

**Figure 1:**
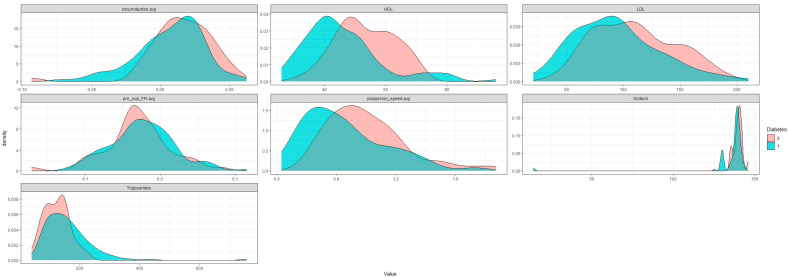
Gait analysis variables analyzed with PODOSmart^®^ that presented statistically significant difference (*p* < 0.05) among the study groups. pro: pronation; sup: supination; HO: Heel off; TO: Toe off; avg: average; SD: standard deviation.

Figure 1 summarizes the parameters measured with PODOSmart^®^ during free gait that were significantly different between the T2DM and HC groups. The parameters were significantly lower in T2DM patients compared with HC, including stride length (*p* < 0.001), walking speed (*p* < 0.001), angle attack (*p* < 0.001), oscillation speed (*p* < 0.001), time digitigrade phase (*p* = 0.007), pronation-supination toe off (*p* = 0.008) and clearance (*p* = 0.019). 

The comparison of all measured parameters with PODOSmart^®^ during free gait are provided in Appendix A Table A1. 

**Figure 2:**
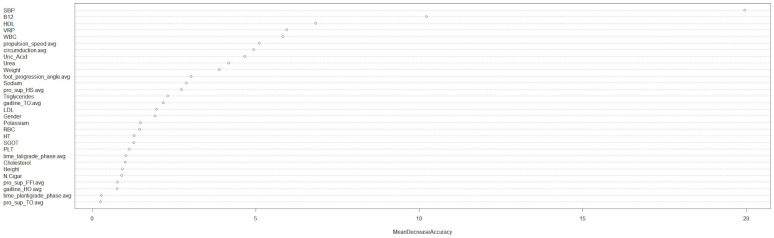
Random Forest model exploring the anthropometric, biochemical and gait factors associated with T2DM. MeanDecreaceAccuracy: Mean Decrease Accuracy plot expresses how much accuracy the model loses by excluding each variable. The more the accuracy suffers, the more important the variable is to the successful classification. The variables are presented from descending importance. SBP, Systolic blood pressure; HDL, high-density lipoprotein; VRP, virtual-repellent-point; WBC, white blood cells; pro, pronation; sup, supination; HS, high step; TO, toe off; LDL, low-density cholesterol; RBC, red blood cells; HT, hematocrit; SGOT, serum glutamic-oxaloacetic transaminase; PLT: platelets; N.Cigar: number of cigars; FFI, Flat foot in; and HO, heal off.

The Random Forest machine learning model was used to determine the important factors (anthropometric, biochemical, dietary and gait analysis) for the prediction of T2DM. The model presented good accuracy (0.857) and high precision (AUC: 0.94; 95% CI: 0.69, 0.95). The Mean Decrease Accuracy plot was created to express how much accuracy the model loses by excluding each variable (Figure 2). The more the accuracy suffers, the more important the variable is to the successful classification. The variables are presented from descending importance. According to the model, (a) from the anthropometrics, an increased systolic blood pressure and body weight; (b) from the biochemical, increased B12, low HDL, high WBC, uric acid and urea, triglycerides and CRP; and (c) from the gait parameters, slower propulsion speed and circumduction, foot progression angle, pronation-supination toe off and gaitline toe off were the most predictive for the occurrence of T2DM (Figure 2). 

## 4. Discussion

In the present study, T2DM patients without clinical signs of DPN had significantly higher cardiometabolic parameters (e.g., weight, BMI, WC, glucose, HbA1c, SBP, uric acid and lower HDL-C) compared with HC that were significantly correlated with alterations in various gait parameters including stride length, walking speed, angle attack, oscillation speed, time digitigrade phase, pronation-supination toe off and clearance. The dietary habits were similar between T2DM and HC participants, showing moderate adherence to the Mediterranean diet.

Our findings highlight the presence of early gait changes in T2DM patients in comparison to age-matched HC. Nevertheless, T2DM participants, in the majority, presented low diabetes distress, indicative of their good quality of life, the relevantly good diabetes control and the presence of minimal disease complications. Previous studies have evaluated gait abnormalities in T2DM individuals mainly in relation to the presence of DPN [26]. For example, T2DM patients complicated with DPN possessed shorter stride length, lower gait velocity, longer stride and stance time, as well as higher maximum knee extension moment compared with T2DM patients without DPN, as reported in a recent meta-analysis [2]. Indeed, gait disorders in T2DM have been linked to both muscle weakness and sensory loss, as well as with neurological dysfunction [27]. Since gait abnormalities can lead to increased risks of fall, foot ulceration and low quality of life in T2DM, it is clinically important to promote strategies to efficiently prevent gait changes [28,29]. The present study supports that gait may be impaired early in the course of T2DM, even in the absence of DPN, highlighting the need for performing gait analysis long before the development of DPN. 

Quantitative gait analysis technology providing accurate and reliable gait measurements has been developed [7]. In this context, PODOSmart^®^ insoles were designed for gait analysis parameter measurement, representing an easy to perform, reliable and repeatable method [30]. The present study used PODOSmart^®^ insoles to evaluate gait changes in T2DM and HC individuals. Of note, wearable gait analysis systems measuring kinematic data, spatial and temporal parameters, have also been evaluated in T2DM patients [31]. In patients with T2DM and impaired glucose tolerance, ankle strength may decrease during walking, which is due to weakness of the peripheral forces of the sole and ankle [32]. Studies suggest that gait dysfunction occurs when attentional regulatory mechanisms are required to control movement, long before sensory information from the foot is lost [33].

Furthermore, our results suggest an association between parallel T2DM risk and body weight gain, which is consistent with previous studies [34,35]. Weight management is essential for better glycemic control [36]. Body weight and body composition have previously been associated with changes in gait parameters in obesity [37]. A common phenomenon in the elderly is the coexistence of increased body weight and sarcopenia [38] affecting gait abnormalities, such as stride length and gait line length [39].

In the present study, the MedDiet score was moderate in both T2DM patients and HC. Adherence to the MedDiet has been previously positively associated with physical strength [17], better HbA1c control [40] and gait speed in T2DM patients [17,41]. However, in our study, we could not detect correlations of MedDiet with gait parameters due to the similar dietary profile of our study participants; therefore, nor the overall score nor any of the Mediterranean score factors used was correlated with gait characteristics.

The present study has several strengths, including the performance of gait analysis in T2DM patients without DPN and the use of a machine random forest learning model to accurately predict early gait changes associated with cardiometabolic complications in T2DM. However, there are limitations. The sample size was appropriately small and had homogeneity in disease burden and dietary habits. Therefore, we were unable to determine early potential quality of life complications related to gait changes or dietary factors that could be used in future dietary interventions to prevent these early diabetic complications.

## 5. Conclusions

T2DM patients without DPN can experience significant gait abnormalities compared with healthy individuals. Taking into consideration that gait impairment may lead to falls and injuries, thus reducing individual’s quality of life, it is important to early diagnose and efficiently prevent gait changes in T2DM patients. 

## Figures and Tables

**Table 1:** Study participants’ demographic, anthropometric and clinical characteristics.

Variables	T2DM Group	HC Group	*p*-Value
Gender, n	78F/21M	34F/16M	0.78
Age, years (mean ± SD)	66.4 ± 11.5	62.1 ± 7.9	0.657
Height, m (mean ± SD)	1.7 ± 0.1	1.7 ± 0.1	0.564
Weight, kg (median, MAD)	85.0 (13.3)	75.5 (15.6)	<0.001
BMI, kg/m^2^ (median, MAD)	30.5 (4.4)	27.5 (6.0)	<0.001
WC, cm (mean ± SD)	103 ± 17	93 ± 19	<0.001
SBP, mmHg (median, MAD)	131.0 (13.3)	120 (5.9)	<0.001
Glucose, mg/dL (median, MAD)	130.0 (25.2)	88.0 (9.6)	<0.001
HBA1c, % (median, MAD)	6.5 (0.7)	4.6 (0.3)	<0.001
Total Cholesterol, mg/dL (mean ± SD)	176 ± 4	196 ± 35	0.002
HDL-C, mg/dL (median, MAD)	43 (10)	51 (10)	<0.001
LDL-C, mg/dL (median, MAD)	90 (40)	107 (44)	0.007
Triglycerides, mg/dL (median, MAD)	148 (68)	126 (44)	0.005
Sodium, mEq/L (median, MAD)	139.0 (1.5)	140.0 (1.5)	<0.001
WBC, 10^9^/L (median, MAD)	7.56 (2.00)	6.70 (1.36)	<0.001
CRP, mg/dL (median, MAD)	0.20 (0.03)	0.22 (0.01)	<0.001
Uric Acid, mg/dL (median, MAD)	5.6 (1.7)	4.3 (0.8)	<0.001
Urea, mg/dL (median, MAD)	34.5 (9.6)	31 (5.9)	<0.001
Creatinine, mg/dL (median, MAD)	0.9 (0.2)	0.8 (0.2)	0.002

T2DM: type 2 diabetes mellitus; HC: healthy controls; BMI: Body Mass Index; WC: Waist Circumference; SBP: Systolic Blood Pressure; HDL-C: High Density Cholesterol; LDL-C: Low Density Cholesterol; WBC: White Blood Cells; and CRP:C-reactive protein. Values are Mean (±Standard Deviation) or Median (MAD) in non-normally distributed variables; mEq/L: milliequivalents per liter; mg/dL: milligrams/deciliter.

**Table 2:** Dietary habits relevant to the adherence to the Mediterranean Diet Pattern in both study groups.

Food Type	T2DM Mean (±SD)	HC Mean (±SD)	*p*-Value
Potatoes	1.66 (±0.79)	1.86 (±0.53)	0.790
Whole grains	1 (±1.48)	1 (±1.48)	0.078
Olive oil	5 (±0)	5 (±0)	0.115
Vegetables	2 (±1.93)	2 (±2.08)	0.118
Full Fat Dairies	4.95 (±0.22)	4.9 (±0.3)	0.251
Fruits	2 (±0)	2 (±0)	0.339
Alcohol	5 (±0)	5 (±0)	0.380
White meat	5 (±0)	5 (±0)	0.521
Red meat	4.61 (±0)	4.55 (±0)	0.618
Legumes	2 (±0)	2 (±0)	0.868
Fish/shelfish	1 (±0)	1 (±0)	0.888
MedDiet Score	34 (±3)	34 (±1)	0.718

T2DM: type 2 diabetes mellitus; HC: healthy controls.

## Data Availability

The raw data supporting the conclusions of this article will be made available by the authors, without undue reservation.

## Appendix A

**Table A1:** Gait analysis variables analyzed with PODOSmart^®^.

Variables	T2DM	HC	*p*-Value
stride_length.avg	1.04 (±0.13)	0.94 (±0.15)	**0.000**
walking_speed.avg	2.96 (±0.52)	2.64 (0.54)	**0.001**
angle_attack.avg	0.3 (±0.07)	0.26 (±0.09)	**0.001**
oscillation_speed.avg	1.33 (±0.17)	1.23 (±0.18)	**0.002**
time_digitigrade_phase.avg	279.09 (±37.21)	259.98 (±41.77)	**0.007**
pro_sup_TO.avg	meion 0.03 (±0.07)	0 (±0.07)	**0.009**
clearance.avg	11.97 (±6.3)	9.48 (±5.98)	**0.020**
time_plantigrade_phase.avg	367.81 (±102.8)	338.81 (121.86)	0.151
pro_sup_FFI.avg	MEION 0.03 (0.04)	MEION 0.02 (±0.04)	0.393
pro_sup_HO.avg	0.03 (±0.05)	0.03 (±0.07)	0.501
cadence.avg	47.31 (±4.9)	46.94 (±4.96)	0.667
flight_time.avg	490.57 (±46.512	489.27 (±55.46)	0.887
gaitline_TO.avg	3 (0)	2.99 (0.02)	**<0.001**
propulsion_speed.avg	0.81 (0.26)	0.94 (0.26)	**<0.001**
foot_progression_angle.avg	0.19 (0.11)	0.12 (0.13)	**0.006**
time_taligrade_phase.avg	148.93 (108.95)	117.35 (60.34)	**0.016**
circumduction.avg	0.01 (0.02)	0.02 (0.02)	**0.039**
gaitline_TS.avg	2.74 (0.2)	2.67 (0.21)	0.339
pro_sup_HS.avg	MEION 0.12 (0.06)	MEION 0.12 0.06)	0.404
gaitline_HS.avg	2.18 (0.44)	2.05 (0.47)	0.482
Confusions	75 (13.34)	78 (10.38)	0.825
gaitline_HO.avg	2.77 (0.33)	2.82 (0.25)	0.939

Variables that presented statistically significant differences (*p* < 0.05) among the T2DM and HC groups are indicated with bold letters. Values are Mean (±Standard Deviation) (*t*-test) or Median (MAD) in non-normally distributed variables.
